# Enrichment of anaerobic nitrate-dependent methanotrophic ‘*Candidatus* Methanoperedens nitroreducens’ archaea from an Italian paddy field soil

**DOI:** 10.1007/s00253-017-8416-0

**Published:** 2017-08-04

**Authors:** Annika Vaksmaa, Simon Guerrero-Cruz, Theo A. van Alen, Geert Cremers, Katharina F. Ettwig, Claudia Lüke, Mike S. M. Jetten

**Affiliations:** 1 Department of Microbiology, IWWR, Radboud University Nijmegen, Nijmegen, The Netherlands; 2 Department of Biotechnology, Delft University of Technology, Delft, The Netherlands; 3 Soehngen Institute of Anaerobic Microbiology, Nijmegen, The Netherlands

**Keywords:** ‘*Candidatus* Methanoperedens nitroreducens’, Anaerobic oxidation of methane, NC10 phylum bacteria

## Abstract

Paddy fields are a significant source of methane and contribute up to 20% of total methane emissions from wetland ecosystems. These inundated, anoxic soils featuring abundant nitrogen compounds and methane are an ideal niche for nitrate-dependent anaerobic methanotrophs. After 2 years of enrichment with a continuous supply of methane and nitrate as the sole electron donor and acceptor, a stable enrichment dominated by ‘*Candidatus* Methanoperedens nitroreducens’ archaea and ‘*Candidatus* Methylomirabilis oxyfera’ NC10 phylum bacteria was achieved. In this community, the methanotrophic archaea supplied the NC10 phylum bacteria with the necessary nitrite through nitrate reduction coupled to methane oxidation. The results of qPCR quantification of 16S ribosomal RNA (rRNA) gene copies, analysis of metagenomic 16S rRNA reads, and fluorescence in situ hybridization (FISH) correlated well and showed that after 2 years, ‘*Candidatus* Methanoperedens nitroreducens’ had the highest abundance of (2.2 ± 0.4 × 10^8^) 16S rRNA copies per milliliter and constituted approximately 22% of the total microbial community. Phylogenetic analysis showed that the 16S rRNA genes of the dominant microorganisms clustered with previously described ‘*Candidatus* Methanoperedens nitroreducens ANME2D’ (96% identity) and ‘*Candidatus* Methylomirabilis oxyfera’ (99% identity) strains. The pooled metagenomic sequences resulted in a high-quality draft genome assembly of ‘*Candidatus* Methanoperedens nitroreducens Vercelli’ that contained all key functional genes for the reverse methanogenesis pathway and nitrate reduction. The diagnostic *mcrA* gene was 96% similar to ‘*Candidatus* Methanoperedens nitroreducens ANME2D’ (WP_048089615.1) at the protein level. The ‘*Candidatus* Methylomirabilis oxyfera’ draft genome contained the marker genes *pmoCAB*, *mdh*, and *nirS* and putative NO dismutase genes. Whole-reactor anaerobic activity measurements with methane and nitrate revealed an average methane oxidation rate of 0.012 mmol/h/L, with cell-specific methane oxidation rates up to 0.57 fmol/cell/day for ‘*Candidatus* Methanoperedens nitroreducens’. In summary, this study describes the first enrichment and draft genome of methanotrophic archaea from paddy field soil, where these organisms can contribute significantly to the mitigation of methane emissions.

**Electronic supplementary material:**

The online version of this article (doi:10.1007/s00253-017-8416-0) contains supplementary material, which is available to authorized users.

## Introduction

The methane concentration in the atmosphere has increased continuously over the last 150 years. Methane is the second most abundant greenhouse gas and exhibits radiative forcing up to 34 times higher than that of CO_2_ (Myhre et al. 2013). Paddy fields are a significant source of methane and contribute 10–20% to global methane emissions (Bodelier 2011; Conrad 2009). The cultivated area dedicated to rice agriculture occupies approximately 160 million ha of land worldwide and is predicted to increase by 60% in the coming decades. Without changes in cultivation practices, such increases will result in even higher atmospheric methane emissions.

The global biogenic methane budget is directly linked to the activity of methanogenic and methanotrophic microorganisms in the environment. Methanotrophic organisms function as a biofilter, and without their contribution, it is estimated that the atmospheric methane concentration would be 10–60% higher (Conrad 2009). Whereas aerobic methanotrophs are well studied, much less is known about methane removal in oxygen-limited nitrogen-loaded freshwater environments. NC10 phylum (‘*Candidatus* Methylomirabilis oxyfera’) bacteria and ‘*Candidatus* Methanoperedens nitroreducens’ archaea are the only methanotrophic microorganisms known to directly couple the anaerobic oxidation of methane to the nitrogen cycle*.* NC10 phylum bacteria use nitrite as an electron acceptor (A), and ‘*Candidatus* Methanoperedens nitroreducens’ archaea perform nitrate reduction (B) with methane as an electron donor according to the following reactions:

$$ 8{{\mathrm{N}\mathrm{O}}_2}^{-}+3{CH}_4+8{\mathrm{H}}^{+}\to 4{\mathrm{N}}_2+3{CO}_2+10{\mathrm{H}}_2\mathrm{O}\ \left(\mathrm{A},\Delta G{0}^{\prime }=-987\ \mathrm{kJ}/\mathrm{mol}\ {CH}_4\right) $$

$$ 2\mathrm{x}\ \left(4{{\mathrm{NO}}_3}^{-}+{CH}_4\to 4{{\mathrm{NO}}_2}^{-}+{CO}_2+2{\mathrm{H}}_2\mathrm{O}\right)\ \left(\mathrm{B},\Delta G{0}^{\prime }=-503\ \mathrm{kJ}/\mathrm{mol}\right) $$

$$ 8{HNO}_3+5{CH}_4\to 5{CO}_2+4{\mathrm{N}}_2+14{\mathrm{H}}_2\mathrm{O}\ \left(\mathrm{sum}\right) $$

In 2006, an enrichment culture in which nitrate and nitrite reduction were coupled to the anaerobic oxidation of methane was described for the first time (Raghoebarsing et al. 2006). In that study, an inoculum from the sediment of a freshwater canal was used to start an anaerobic enrichment. After 16 months, the culture was dominated by a consortium consisting of archaea (10–15% of cells) belonging to the *Methanosarcinales* family that were only distantly related to ANME2D (86–87% in 16S ribosomal RNA (rRNA identity)) and a bacterium (approximately 80% of cells) of the candidate division NC10*.* The enriched co-culture preferred nitrite over nitrate as the substrate, although activity was observed with both substrates (Raghoebarsing et al. 2006). The nitrite-dependent anaerobic oxidation of methane (AOM) was later assigned to phylum NC10 bacteria, which are able to carry out this process in the absence of other microorganisms (Ettwig et al. 2008). The bacterium uses an intra-aerobic mechanism in which oxygen is produced via a putative nitric oxide dismutase and subsequently used for methane oxidation via the particulate methane monooxygenase complex. Assembly of the genome of the NC10 bacterium revealed a complete methane oxidation pathway that included the *pmoCAB* operon and an incomplete denitrification pathway. It was hypothesized that the dismutation of nitric oxide to oxygen and nitrogen supplies O_2_ for the methane monooxygenase. These NC10 phylum bacteria were named ‘*Candidatus* Methylomirabilis oxyfera’ (Ettwig et al. 2010). Sequencing of the genome of the AOM archaea and identification of their nitrate reductase indicated that these archaea could couple nitrate reduction to AOM (Arshad et al. 2015; Haroon et al. 2013). The responsible archaea were named ‘*Candidatus* Methanoperedens nitroreducens.’

Recent microbial ecology studies have indicated sufficient presence and activity of methanotrophic archaea in paddy field soils (Lee et al. 2015; Vaksmaa et al. 2016) to warrant investment in a long-term enrichment procedure to obtain these paddy field AOM archaea and to study their physiology and metabolic potential in more detail.

To achieve this goal, we started a sequencing batch bioreactor continuously fed with nitrate and methane and inoculated with soil from an Italian paddy field soil harboring substantial AOM archaeal cell numbers (Vaksmaa et al. 2016). After establishing nitrate-dependent methane oxidation, the total DNA of this biomass was sequenced using Ion Torrent technology, and the draft genome was annotated and analyzed. The enriched microbial community was further characterized by microscopy, ^13^CH_4_ and ^15^N activity assays, and qPCR.

## Materials and methods

### Source of inoculum

The soil was sampled in September 2013 from paddy fields at the Italian Rice Research Unit in Vercelli, Italy (08° 22′ 25.89″ E; 45° 19′ 26.98″ N). These fields of silt loam soil were flooded with approximately 15 cm of water and regularly tilled. The soil of the experimental field was fertilized with 147.5 kg/ha nitrogen and 183 kg/ha potassium 21 days after flooding. Soil was collected 95 days after flooding. The rice variety cultivated in the field plots was *Oryza sativa japonica* Onice. The soil was sampled down to 20 cm and transported to the laboratory in a container flooded with water sampled from the field. After storage at 4 °C for 6 months, the reactor was started with 200 g of soil (wet weight).

### Enrichment culture

A 2-L bioreactor (Applikon, The Netherlands) was operated at 27 °C as a sequencing batch reactor. The sequence consisted of 12 h cycles of 10 h of constant medium supply, 1 h of biomass settling, and 1 h of pumping out of excess liquid. The medium contained CaCl_2_·2H_2_O (0.15 g/L) and KH_2_PO_4_ (0.01 g/L) and was autoclaved before the addition of MgSO_4_·7H_2_O (0.1 g/L); 5 mL of a trace element stock solution composed of ZnSO_4_·7H_2_O (0.2875 g/L), CoCl_2_·6H_2_O (0.12 g/L), CuSO_4_ (0.8 g/L), NiCl_2_·6H_2_O (0.19 g/L), H_3_BO_3_ (0.014 g/L), MnCl_2_·4H_2_O (0.2 g/L), Na_2_WO_4_·2H_2_O (0.02 g/L), Na_2_MoO_4_·2H_2_O (0.0968 g/L), SeO_2_ (0.027 g/L), and CeCl_2_ (0.023 g/L); 3 mL of an iron stock solution composed of FeSO_4_·7H_2_O (5 g/L) and NTA (10.31 g/L); and 1 mL of vitamin solution (DSMZ 141). The medium was constantly sparged with Ar/CO_2_ (95:5%) to maintain anaerobic conditions prior to being supplied by peristaltic pump to the bioreactor at a flow rate of 18.75 mL/h. The NaNO_3_ concentration in the medium was increased from 1.25 to 5 mM after a year of operation due to an increased consumption rate. The bioreactor was operated at pH 7, maintained with automatic supply of KHCO_3_, stirred at 150 rpm, and sparged with CH_4_-CO_2_ (95% vol/vol; purity 99.995%; flow rate 4.26 mL/min).

### Activity measurements

Activity measurements were performed in the whole reactor in batch mode after cutoff of the supply of medium and methane. The nitrate in the bioreactor was depleted, and the headspace was flushed with Ar-CO_2_ (95:5). Once residual methane was no longer detected by gas chromatography, 5 mM ^15^N-NaNO_3_ and 20% ^13^C-CH_4_ (vol/vol) were added to the reactor and headspace, respectively. Gas samples of 100 μL were taken at various time points over 3–7 days; the production of ^13^C-CO_2_ was monitored by gas chromatography-mass spectrometry (GC-MS) (Agilent 5975 inert MSD, Agilent, USA), and the consumption of CH_4_ was measured by GC (Hewlett Packard 5890, USA). Liquid culture samples of 1 mL (duplicate) were collected for the determination of NO_3_ (measured by a Sievers 280i NO analyzer, GE Analytical Instruments, USA) and NO_2_ and NH_4_ (measured by colorimetric assays as described by Kartal et al. 2006). Liquid samples were centrifuged for 1 min at 14,000*g*, and the supernatant was removed for storage at −20 °C until analysis.

Further activity assays were performed in 60-mL serum bottles with 15 mL of biomass from the reactor. The reactor was stirred at 500 rpm for 5 min before sampling to ensure appropriate mixing of all settled biomass. After transfer of 15 mL of slurry, fresh medium (composition described above) and ^15^N-NaNO_3_ (final concentration in bottles, 5 mM) or ^15^N-NaNO_2_ (final concentration in bottles, 1 mM) were added. The bottles were made anaerobic by 5 cycles of vacuum and purging with argon-CO_2_ (95–5%). An overpressure of 0.5 bar was introduced to the bottles, and 10% ^13^C-CH_4_ was added. Measurements of ^13^C-CO_2_ and CH_4_ in a 50-μL headspace sample were obtained by GC-MS and GC, respectively. Calibration was performed with standard gas consisting of QS:1.06%:0.82%:1.32%:459 ppm He/CO_2_/N_2_/O_2_/N_2_O (Air Liquide BV, The Netherlands). Analysis of nitrogen compounds was performed as described above for the whole reactor as batch.

### DNA extraction

DNA was extracted from 10 mL of reactor biomass in duplicate using a PowerSoil DNA Isolation Kit (MO BIO Laboratories Inc., Carlsbad, CA, USA) according to the manufacturer’s protocol with addition of 3 min beadbeating step. DNA quantity and quality were assessed by UV-vis spectroscopy (NanoDrop, ND-1000, Isogen Life Science, The Netherlands).

### Quantification by qPCR

The abundances of ‘*Candidatus* Methanoperedens nitroreducens,’ NC10 phylum bacteria, total bacteria, and total archaea were quantified based on 16S rRNA gene amplification by qPCR. The qPCR reactions were performed in triplicate on all DNA extracts. ‘*Candidatus* Methanoperedens nitroreducens’ were targeted with the clade-specific primers 641F (5′ ACTGDTAGGCTTGGGACC3′) and 834R (5′ ATGCGGTCGCACCGCACCTG3′) (previously reported as FISH probes) (Schubert et al. 2011). NC10 phylum bacteria were amplified with the 16S rRNA primers p2F_DAMO (5′ GGGGAACTGCCAGCGTCAAG3′) and p2R_DAMO (5′ CTCAGCGACTTCGAGTACAG3′) (Ettwig et al. 2009b). The total number of archaea was quantified using the following primers: Arch-349F (5′ GYGCASCAGKCGMGAAW3′) and Arch-807R (5′ GGACTACVSGGGTATCTAAT3′). For bacteria, the primers Bac-341F (5′ CCTACGGGNGGCWGCAG3′) and Bac-515R (5′ TTACCGCGGCTGCTGGCAC3′) (Klindworth et al. 2013) were used. All qPCR reactions were performed using PerfeCTa Quanta master mix (Quanta Biosciences, Gaithersburg, MD, USA) and 96-well optical plates on a Bio-Rad IQ™ 5 cycler (Bio-Rad, USA). Absolute quantification was performed by comparison to standard curves obtained using a tenfold serial dilution of pGEM-T Easy plasmid DNA (Promega, USA) carrying an insert of the target gene obtained using the same primers used for qPCR. Standard curve samples were used as a control for each qPCR run.

### Fluorescence in situ hybridization

‘*Candidatus* Methanoperedens nitroreducens’ and NC10 phylum bacteria were detected using 2 mL of reactor biomass sample. The sample was pelleted, washed twice with 1 mL of 1× PBS, and fixed with paraformaldehyde on ice for 3 h. Fluorescence in situ hybridization (FISH) was performed as described by Ettwig et al. (2008).

### Metagenome sequencing

Ion Torrent sequencing was performed on DNA samples obtained from the bioreactor after 1 and 2 years of operation. DNA was isolated as described above. In total, 185 ng of isolated genomic DNA was sheared for 9 min using a Bioruptor® UCD-200 (Thermo Fisher Scientific Inc., USA). Libraries were prepared using an Ion Plus Fragment library kit (Thermo Fisher Scientific Inc., USA) according to the manufacturer’s instructions. For size selection of the adapter ligated fragments, an E-Gel® electrophoresis system was used with a 2% E-Gel® SizeSelect™ agarose gel (Life Technologies, Bleiswijk, The Netherlands). Eight cycles of amplification of the size-selected fragments were performed as suggested in the protocol. The concentrations and fragment lengths of the libraries were determined with a Bioanalyzer® 2100 and High Sensitivity DNA Kit (Agilent Technologies, Santa Clara, CA, USA.) The library was diluted to a final concentration of 26 pM for emulsion PCR. Emulsion PCR was performed using an Ion OneTouch™ 2 Instrument and Ion PGM™ Template OT2 400 Kit (Thermo Fisher Scientific Inc., USA) according to the manufacturer’s instructions. The template-positive Ion Sphere™ Particles (ISPs) were enriched using the Ion One Touch™ ES (Thermo Fisher Scientific Inc., USA), loaded on an Ion 318™ v2 Chip and sequenced using an Ion PGM™ Sequencing 400 Kit with 850 nucleotide flows according to the manufacturer’s instructions. After sequencing, all raw reads were imported into CLC Genomics Workbench v. 9 (QIAGEN Aarhus A/S, Denmark) for initial data analysis, including trimming of low-quality and short reads (cutoff value of 100 nucleotides), followed by assembly of the reads obtained from both sequencing runs (word size 30, bubble size 5000). The raw reads of metagenome sequencing after 1 and 2 years have been deposited to the European Nucleotide Archive, with study accession number PRJEB20370. To extract the contigs of ‘*Candidatus* Methanoperedens nitroreducens Vercelli,’ the contigs were binned based on GC content and coverage using RStudio (RStudio Team 2015) with the GC script. The contigs of ‘*Candidatus* Methanoperedens nitroreducens Vercelli’ were extracted from all assemblies, and reads mapping to contigs were reassembled in CLC (word size 30, bubble size 5000). The completeness of the draft genome and contamination were assessed by CheckM (Parks et al. 2015). MaGe, online full annotation and integration automated pipeline (Vallenet et al. 2009, 2006, 2017), was used to annotate the genome of ‘*Candidatus* Methanoperedens nitroreducens Vercelli,’ and this subsequently was visualized in Artemis (Rutherford et al. 2000). The annotated genome of ‘*Candidatus* Methanoperedens nitroreducens Vercelli’ has been deposited at GenBank under the accession ERS1800110. BLAST was used to search for key genes in ‘*Candidatus* Methylomirabilis oxyfera.’ The contigs from *‘Candidatus* Methylomirabilis oxyfera’ were extracted after differential mapping. The reads from each year were mapped to the assembly from the combined years (0.5 length fraction and 0.95 similarity), and both values for each read were plotted against one another in RStudio. The contigs containing the *nod*, *pmo*, *nirS*, and 16S genes were manually curated to extend them over the ends of the genes. The annotated contigs were checked using the visualization and annotation tool Artemis.

## Results

### Enrichment procedure for anaerobic oxidation of methane organisms in the bioreactor

**Fig. 1:**
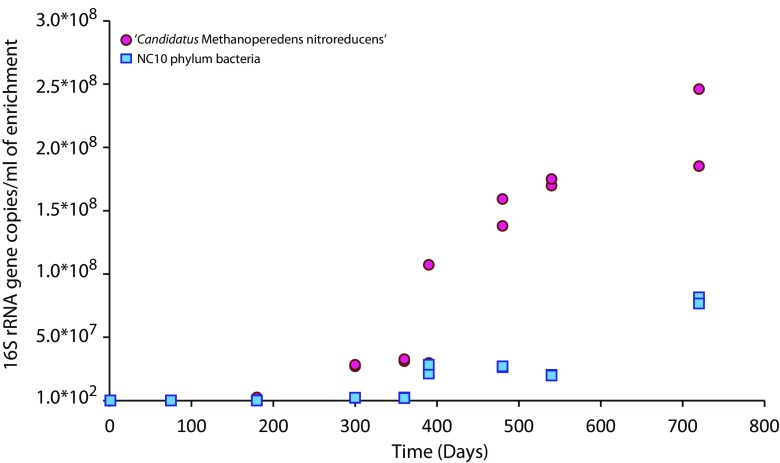
qPCR quantification of the 16S rRNA gene copy numbers of ‘*Candidatus* Methanoperedens nitroreducens’ and NC10 phylum bacteria over the period of 2 years (all time points were analyzed using duplicate DNA extractions and triplicate qPCR reactions). The time in days is depicted horizontally, whereas the 16S rRNA copies per milliliter of enrichment are depicted vertically

The microbial cells in the inoculum and enrichment culture were quantified by qPCR based on the 16S rRNA gene. The abundance of ‘*Candidatus* Methanoperedens nitroreducens’ was one to two orders of magnitude higher than that of NC10 phylum bacteria in the inoculum slurry (Table 1). After 2 years of enrichment, ‘*Candidatus* Methanoperedens nitroreducens’ constituted approximately 22% of the total microbial community based on the qPCR results and was one order of magnitude more abundant than NC10 phylum bacteria. The growth of ‘*Candidatus* Methanoperedens nitroreducens’ started after 10 months of enrichment. The lag phase of NC10 phylum bacteria appeared to be longer, but after a year, their 16S rRNA gene copy numbers had already increased from 10^3^ to 10^7^ per milliliter (Fig. 1).

**Table 1:** 16S rRNA gene copies of total archaea, ‘*Candidatus* Methanoperedens nitroreducens,’ total bacteria, and NC10 phylum bacteria in the enrichment at the start of the reactor and after 0.5, 1, and 2 years of operation (mean ± SE; *n* = 6), calculated per 1 mL of reactor sample

	*T* = 0	0.5 years	1 year	1.5 years	2 years
Total archaea	2.6 ± 0.2 × 10^6^	6.7 ± 0.3 × 10^6^	1.3 ± 0.8 × 10^8^	2.4 ± 0.4 × 10^8^	6.6 ± 0.9 × 10^8^
*M. nitroreducens*	1.9 ± 0.1 × 10^5^	2.7 ± 0.4 × 10^6^	3.2 ± 0.1 × 10^7^	1.7 ± 0.0 × 10^8^	2.2 ± 0.4 × 10^8^
Total bacteria	1.6 ± 0.1 × 10^8^	3.0 ± 1.2 × 10^7^	2.3 ± 0.0 × 10^7^	1.7 ± 0.0 × 10^7^	3.2 ± 0.3 × 10^8^
NC10 phylum bacteria	1.9 ± 0.9 × 10^3^	8.8 ± 4.8 × 10^3^	2.2 ± 0.3 × 10^6^	2.0 ± 0.1 × 10^6^	7.9 ± 0.3 × 10^7^

### Fluorescence in situ hybridization

**Fig. 2:**
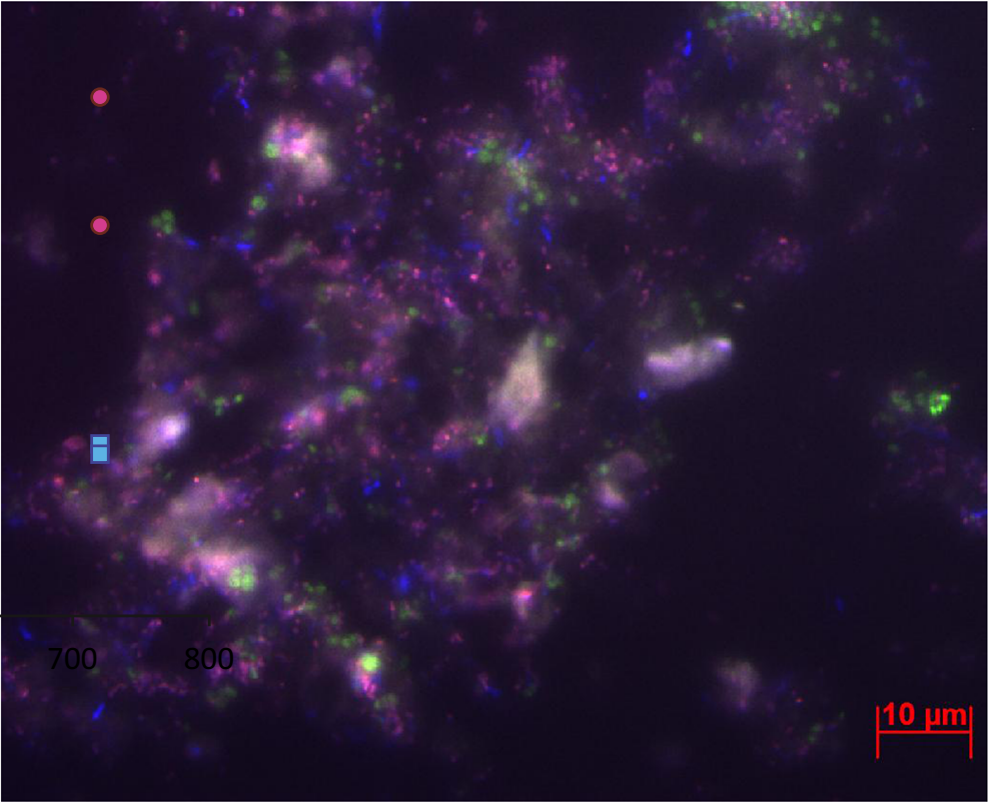
Fluorescent micrograph of biomass sample from the enrichment culture. *Blue* corresponds to Cy5-EUBMIX, total bacteria, *pink* to NC10 phylum bacteria (Cy5-EUBMIX, DAMO193), and *green* to ‘*Candidatus* Methanoperedens nitroreducens’ (FLUOS 641) (Color figure online)

Biomass samples from the enrichment culture were analyzed with specific probes for ‘*Candidatus* Methanoperedens nitroreducens’ and NC10 phylum bacteria after 2 years of enrichment. Both microorganisms were present in the reactor (Fig. 2), although the cell numbers of the NC10 bacteria appeared to be higher than determined by qPCR.

### Activity of the nitrate-dependent anaerobic oxidation of methane co-culture

**Fig. 3:**
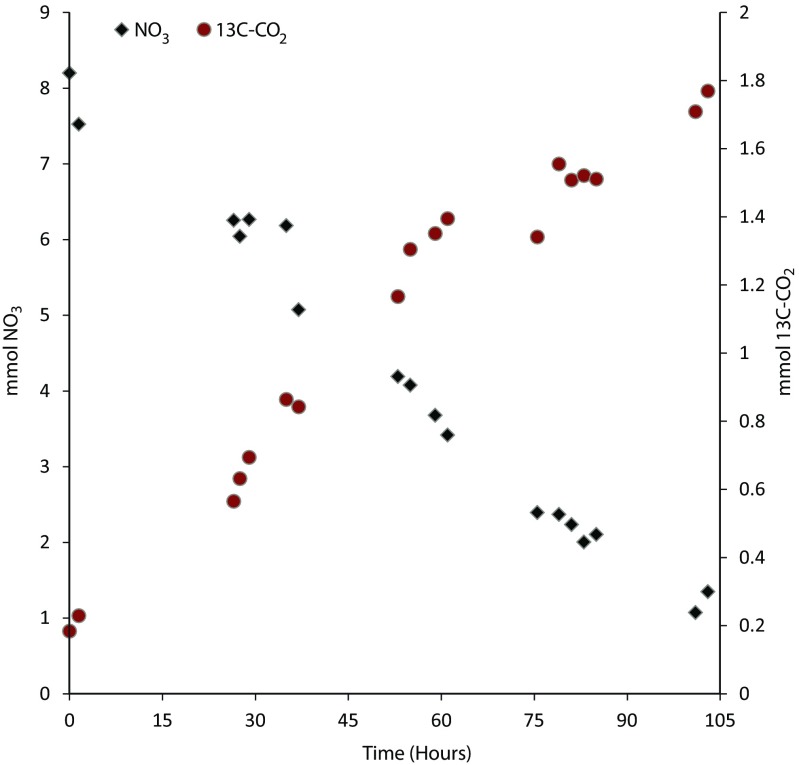
Nitrate consumption and ^13^C-CO_2_ production in batch assays of the total reactor. The time in hours is depicted horizontally, whereas the total amounts in millimole of nitrate (*left axis*) and ^13^C-CO_2_ (*right axis*) are depicted vertically

The culture in the bioreactor oxidized CH_4_ to CO_2_ using nitrate as an electron acceptor. The oxidation rates increased over the time span of 2 years. The initial potential to oxidize methane at the expense of nitrate in the soil slurries was 16.8 nmol/g dry weight/day with 2 mM NaNO_3_ versus 3.7 nmol/g/day in the controls, which were incubated without any external electron acceptor and 10% methane in the headspace (Vaksmaa et al. 2016). During the 2 years of bioreactor operation, neither nitrite (<80 μmol/L) nor ammonia was detected (below the detection level) in significant quantities. After 2 years, the nitrate consumption and methane consumption were 0.055 and 0.012 mmol/h^/^L, respectively (Fig. 3). Activity measurements with ^13^C-CH_4_ with 1 mM nitrite and 5 mM nitrate in serum bottles after 2 years of enrichment indicated that only the conversion of methane to CO_2_ (0.19 mmol/h/L) occurred in bottles amended with nitrate. Surprisingly, the methane conversion rates in serum bottles amended with nitrite were similar to those in the control sample, where no activity was seen (Supplementary Fig. S1).

### Metagenomic analysis and classification based on the 16S ribosomal RNA gene

After 1 and 2 years of enrichment, DNA was extracted from the culture and sequenced by Ion Torrent technology (Supplementary Table S1) to first analyze the 16S rRNA gene composition and then to assemble draft genomes.

**Fig. 4:**
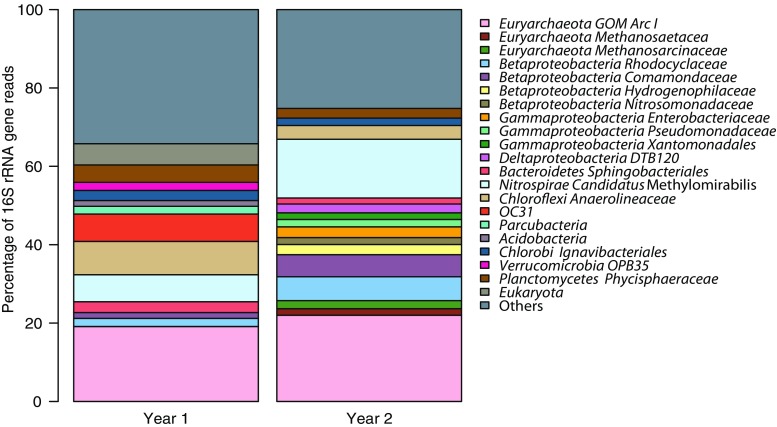
Phylogenetic classification based on 16S rRNA gene reads obtained from the metagenome after enrichment for 1 and 2 years

**Fig. 5:**
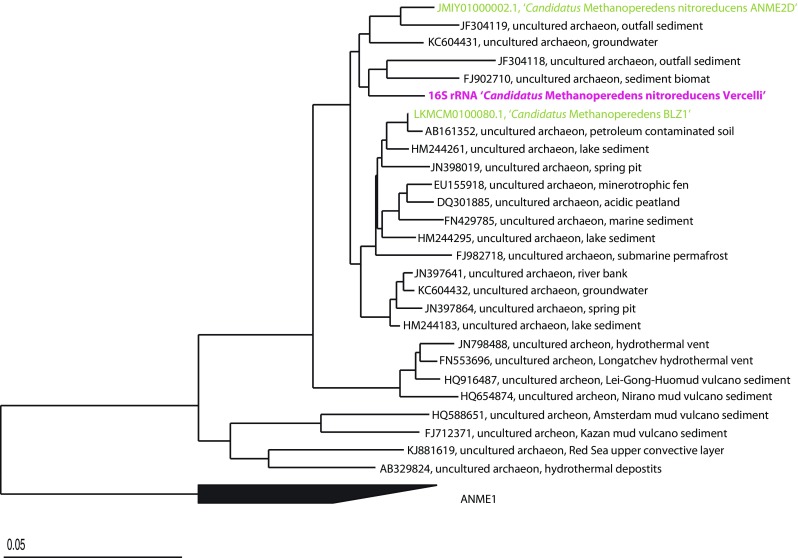
Phylogenetic tree illustrating the relationships between the assembled 16S rRNA contig of ‘*Candidatus* Methanoperedens nitroreducens’ and closely related sequences. The phylogenetic tree was constructed in ARB using the neighbor-joining method. The tree was rooted to the ANME1 cluster. The *scale bar* represents a difference of 0.05 substitutions per site

**Fig. 6:**
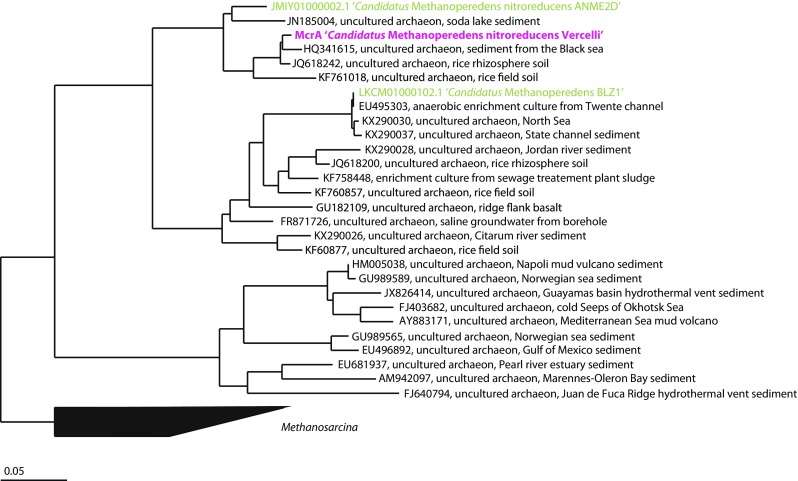
Phylogenetic tree illustrating the relationships between the *mcrA* contig of ‘*Candidatus* Methanoperedens nitroreducens’ and closely related sequences. The phylogenetic tree was constructed in ARB using the neighbor-joining method. The tree was rooted to the *Methanosarcina* cluster, including *Methanosarcina mazei*. The *scale bar* represents a difference of 0.05 substitutions per site

**Fig. 7:**
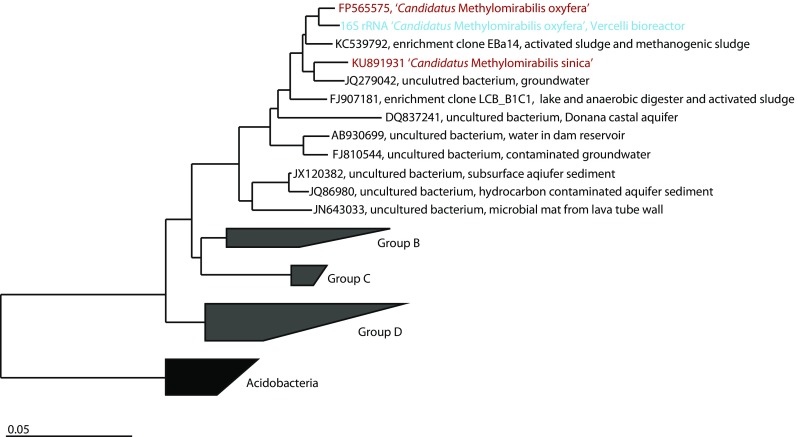
Phylogenetic tree illustrating the relationships between the 16S rRNA contig of Methylomirabilis bacteria from the metagenome and closely related sequences. Depicted is the clustering of the NC10 clade into groups A–D. ‘*Candidatus* Methylomirabilis oxyfera’ and ‘*Candidatus* Methylomirabilis sinica’ of group A are marked in *red*. The *scale bar* represents a difference of 0.05 substitutions per site. The tree was constructed in ARB using the neighbor-joining algorithm with Jukes-Cantor correction. The tree was rooted to *Acidobacteria* (Color figure online)

From the two metagenomes obtained after 1 and 2 years of enrichment, 1014 and 1423, 16S rRNA reads were extracted, respectively. The phylogenetic classifications for groups with an abundance of greater than 1.5% of the total number of 16S rRNA gene reads are shown in Fig. 4. Of the 1014 16S rRNA reads from the first year, 19.1% were assigned to GOM Arc I (the group to which ‘*Candidatus* Methanoperedens’ is classified in the ARB SILVA database (Ludwig et al. 2004)), followed by 8.5% assigned to *Chloroflexi*, 7% OC31, 6.9% *Candidatus* Methylomirabilis (classified as *Nitrospirae* in the ARB SILVA database), and 4.4% *Phycisphaeraceae* of *Planctomycetes*. Of the 1423 reads obtained after 2 years of enrichment, 22% were assigned as GOM Arc I, followed by 15% *Candidatus* Methylomirabilis, 6.1% *Rhodocyclaceae* of *Betaproteobacteria*, 5.6% *Comamonadaceae* of *Betaproteobacteria*, and 3.5% *Anaerolineaceae* of *Chloroflexi*. Draft genomes were binned based on GC content-coverage (Supplementary Fig. S2) and assembled and annotated in MaGe (17). The ‘*Candidatus* Methanoperedens nitroreducens Vercelli’ draft genome contained 250 contigs, with a total size of 3.5 Mb. The completeness as assessed by CheckM was 97.7%. The genome contained all key enzymes for the reverse methane oxidation pathway and nitrate reductase (Supplementary Table S2). Phylogenetic analysis of the 16S rRNA gene from the assembled genome revealed that the ‘*Candidatus* Methanoperedens nitroreducens’ 16S rRNA gene had 96% identity to ‘*Candidatus* Methanoperedens nitroreducens ANME2D’ (JMIY01000002.1) (Haroon et al. 2013) and 97% identity to ‘*Candidatus* Methanoperedens BLZ1’ (LKCM01000080.1) (Arshad et al. 2015) (Fig. 5). The diagnostic methyl-coenzyme M reductase *mcrA* gene showed 96% identity at the protein level to (WP_048089615.1) and 89% identity to (KPQ44219.1) (Fig. 6). The 16S rRNA gene of the NC10 phylum bacteria had 99% nucleotide identity to ‘*Candidatus* Methylomirabilis oxyfera’ (locus tag DAMO__16s_rRNA_1) (FP565575) and clustered within group A of the NC10 phylum (Fig. 7). The ‘*Candidatus* Methylomirabilis oxyfera’ draft genome contained the diagnostic *pmoCAB*, *mxaF* methanol dehydrogenase, *nirS cd1* nitrite reductase, and putative NO dismutase genes. The analyzed *pmoA* gene had 95% identity at the protein level to ‘*Candidatus* Methylomirabilis oxyfera’ (CBE69519). We also identified the methanol dehydrogenase large subunit (*mxaF*), with 85% identity (CBE67248) at the protein level, and two copies of the putative nitric oxide dismutase (*nod*) with 98% identity to CBE69502 and 92% identity to CBE69496. The identified nitrite reductase (*nirS*) had 93% identity to ‘*Candidatus* Methylomirabilis oxyfera’ (CBE69462).

## Discussion

Nitrate-dependent anaerobic oxidation of methane (N-AOM) was discovered a decade ago, but the characterization of the metabolism has been hindered by the slow growth of the responsible organisms. The N-AOM microorganisms ‘*Candidatus* Methanoperedens nitroreducens’ archaea and NC10 phylum bacteria have been detected in various fresh water sediments (Welte et al. 2016). In this study, we started an enrichment culture fed solely with methane and nitrate using a paddy field soil harboring significant amounts of ‘*Candidatus* Methanoperedens nitroreducens’ (Vaksmaa et al. 2016) as the inoculum. Based on qPCR, FISH, and metagenome analyses, the enrichment was dominated by ‘*Candidatus* Methanoperedens nitroreducens’ after 2 years of enrichment.

Many previous enrichments were fed with nitrite or a mixture of nitrite and nitrate instead of nitrate only; such conditions are presumably advantageous to NC10 phylum bacteria. We intentionally omitted ammonium from the medium as other studies showed that such cultures would yield a mixed culture of ‘*Candidatus* Methanoperedens nitroreducens’ and anammox bacteria, which could outcompete NC10 phylum bacteria for nitrite (Shi et al. 2013). Our previous field work demonstrated a high abundance of ‘*Candidatus* Methanoperedens nitroreducens’ in the paddy field soil (Vaksmaa et al. 2016), which was confirmed by qPCR of the inoculum slurry. The inoculum slurry contained approximately 1.9 ± 0.1 × 10^5^ copies per milliliter of the 16S rRNA gene of ‘*Candidatus* Methanoperedens nitroreducens’ and 1.9 ± 0.3 × 10^3^ copies per milliliter of NC10 phylum bacteria. After 2 years of enrichment, these numbers had increased to 2.2 ± 0.4 × 10^8^ 16S rRNA copies per milliliter of ‘*Candidatus* Methanoperedens nitroreducens’, corresponding to 22% of the total detected 16S rRNA copies (bacteria plus archaea). These numbers indicate a doubling time of 1 to 2 months. The abundance of 16S rRNA gene copies of NC10 phylum bacteria was 7.9 ± 0.3 × 10^7^, corresponding to approximately 4% of the total copies.

The qPCR copy numbers correlated well with the metagenome sequencing results for ‘*Candidatus* Methanoperedens nitroreducens’, with an abundance of 16S rRNA reads of 22% after 2 years. The percentage of reads assigned to NC10 phylum bacteria was 15%, possibly indicating underestimation by qPCR. The results based on the two methods presented here provided insight into the growth dynamics of both methane oxidizers. The growth of ‘*Candidatus* Methanoperedens nitroreducens’ was observed after approximately 10 months of acclimatization, whereas for NC10 phylum bacteria, more than a year was necessary before a substantial increase in cell numbers was observed. The initial growth of NC10 phylum bacteria was presumably nitrite-limited. Similar lag phases of the growth of NC10 phylum bacteria in enrichment cultures have been reported previously. Zhu et al. showed that ‘*Candidatus* Methanoperedens nitroreducens’ only started to increase in an enrichment obtained from minerotrophic peatland after 9 months, when significant methane oxidation rates (9 nmol/day/g in serum bottles, based on CO_2_ production) indicated microbial growth (Zhu et al. 2012). In addition to substrate preference and availability, temperature has been implicated as a decisive factor in the outcome of AOM enrichments. In enrichments started from wastewater treatment plant sludge and lake sediments, a co-enrichment of NC10 phylum bacteria and ‘*Candidatus* Methanoperedens nitroreducens’ was obtained at 35 °C, whereas at 22 °C, only NC10 phylum bacteria were enriched (Hu et al. 2009).

In our enrichment culture, the methane oxidation potential increased in accordance with the 16S rRNA copy number. The batch incubations performed with the whole bioreactor revealed average methane oxidation and nitrate reduction after 2 years of 0.055 and 0.012 mmol/h/L, respectively. Based on ^13^C-CO_2_ production, the cell-specific methane oxidation rates after 2 years were 0.57 fmol/cell/day for ‘*Candidatus* Methanoperedens nitroreducens’. This is in the same range as a previously reported nitrate-dependent AOM rates that we measured in paddy field soil in which the estimated cell-specific rates were 1.2 fmol/cell/day of CH_4_ (Vaksmaa et al. 2016) as well as NC10 phylum bacteria enrichment in which the cell-specific rates were about 0.2 fmol/cell/day of CH_4_ (Ettwig et al. 2009b) and is also comparable to rates reported for sulfate-dependent AOM by ANMEs (0.7 fmol CH_4_/cell/day) (Nauhaus et al. 2005). Unfortunately, we did not observe nitrite-dependent methane oxidation in the batch incubation, suggesting that either 1 mM nitrite was greater than the inhibitory concentration for the organism, regardless of their capacity to metabolize nitrite or the biomass requires a longer adaptation time to overcome the previous nitrite limitation. Based on the stoichiometry of the reactions for nitrate- and nitrite-dependent anaerobic oxidation of methane, AOM organisms accounted for approximately 46% of nitrate consumption, whereas presumably other nitrate reducers in the reactor, such as denitrifiers, were responsible for the remaining 54% of nitrate loss.

Metagenome analysis revealed that only a few phyla other than ‘*Candidatus* Methanoperedens nitroreducens’ and NC10 phylum bacteria were represented in greater than 5% abundance. *Anaerolineales* (8.5% abundance) belonging to *Chloroflexi* are obligate anaerobes that have previously been observed in anaerobic methanotrophic (Ettwig et al. 2009a; Siniscalchi et al. 2015) and methanogenic enrichment cultures (Gray et al. 2011; Liang et al. 2015; Yamada et al. 2005). *Anaerolineales* may be responsible for the degradation of *n*-alkanes and release formate, acetate, hydrogen, and carbon dioxide. Hug et al. indicated that *Anaerolineales* may provide organic acids to other microorganisms such as acetoclastic methanogens (DeSantis et al. 2006). The physiology of Candidate division OC31 (7% abundance), which was discovered more recently, remains unknown. *Phycisphaerae*, a class of *Planctomycetes* (4.4% abundance), has also been shown to degrade heteropolysaccharides (Wang et al. 2015) and was previously found to be highly abundant in AOM and other anaerobic enrichment cultures. After 2 years of enrichment, *Rhodocyclaceae* accounted for 6.1% and *Comamonadaceae* for 5.6%. Both of these belong to *Betaproteobacteria*. Members of *Comamonadaceae* can perform denitrification, which may explain the observed nitrate reduction rate, which was higher than expected based on the methane oxidation rate alone.

The 3.5-Mb size of the draft genome of ‘*Candidatus* Methanoperedens nitroreducens Vercelli’ is comparable to those of the publicly available genomes of ‘*Candidatus* Methanoperedens BLZ1’ (3.7 Mb) and ‘*Candidatus* Methanoperedens nitroreducens ANME2D’ (3.2 Mb). The GC content of the ‘*Candidatus* Methanoperedens nitroreducens Vercelli’ genome is 44.1% and is more similar to that of ‘*Candidatus* Methanoperedens nitroreducens ANME2D’ (GC content 43.2%) than ‘*Candidatus* Methanoperedens BLZ1’ (40.8%). Functional gene analysis revealed that the *mcrA* gene has 96% identity to ‘*Candidatus* Methanoperedens nitroreducens ANME2D’ and 89% identity to ‘*Candidatus* Methanoperedens BLZ1’ at the protein level. A similar trend was observed for the majority of enzymes in the reverse methanogenesis pathway. Analysis of the denitrification pathway revealed the presence of nitrate reductases as well as nitric and nitrous oxide reductases in the draft genome, whereas no nitrite reductase could be identified.

In summary, this is the first enrichment culture from paddy field soil supplied solely with nitrate and methane to enrich ‘*Candidatus* Methanoperedens nitroreducens’ and NC10 phylum bacteria. The newly enriched co-culture will be used in future studies to unravel the ecophysiological properties of AOM microbes and investigate their role in mitigating methane emissions from paddy fields.

## Electronic supplementary material

ESM 1 (PDF 512 kb)
