# Supplementary material for: Enrichment of anaerobic nitrate-dependent methanotrophic ‘Candidatus Methanoperedens nitroreducens’ archaea from an Italian paddy field soil
Source: Appl Microbiol Biotechnol. 2017 Aug 4;101(18):7075–84. doi: 10.1007/s00253-017-8416-0 (PMC5569662; doi:10.1007/s00253-017-8416-0)
Supplement: Supplementary file 1 — (PDF 512 kb) [file 253_2017_8416_MOESM1_ESM.pdf]

Applied Microbiology and Biotechnology

Enrichment of anaerobic nitrate-dependent methanotrophic '*Candidatus* Methanoperedens nitroreducens' archaea from an Italian paddy field soil

Annika Vaksmaa[1], Simon Guerrero-Cruz[1], Theo A. van Alen[1], Geert Cremers[1], Katharina F. Ettwig[1], Claudia Lüke[1], Mike S. M. Jetten[1,2,3]

[1]Department of Microbiology, IWWR, Radboud University Nijmegen, Nijmegen, the Netherlands

[2]Department of Biotechnology, Delft University of Technology, Delft, the Netherlands

[3]Soehngen Institute of Anaerobic Microbiology, Nijmegen, the Netherlands

Running Head: Enrichment *of* '*Candidatus* Methanoperedens nitroreducens'

Corresponding Author: Annika Vaksmaa (avaksmaa@science.ru.nl)

Supplementary Figure S1: Formation of ^{13}C-CO_2 in serum batch activity assays with 1 mM nitrite and 10% ^{13}C-CH_4; 5 mM nitrate and 10% ^{13}C-CH_4; 10% ^{13}C-CH_4 only; and without additions. Each treatment was performed in triplicate, and the average is presented in the graph. The time in hours is depicted horizontally, whereas the fraction of ^{13}C-CO_2 is depicted vertically.

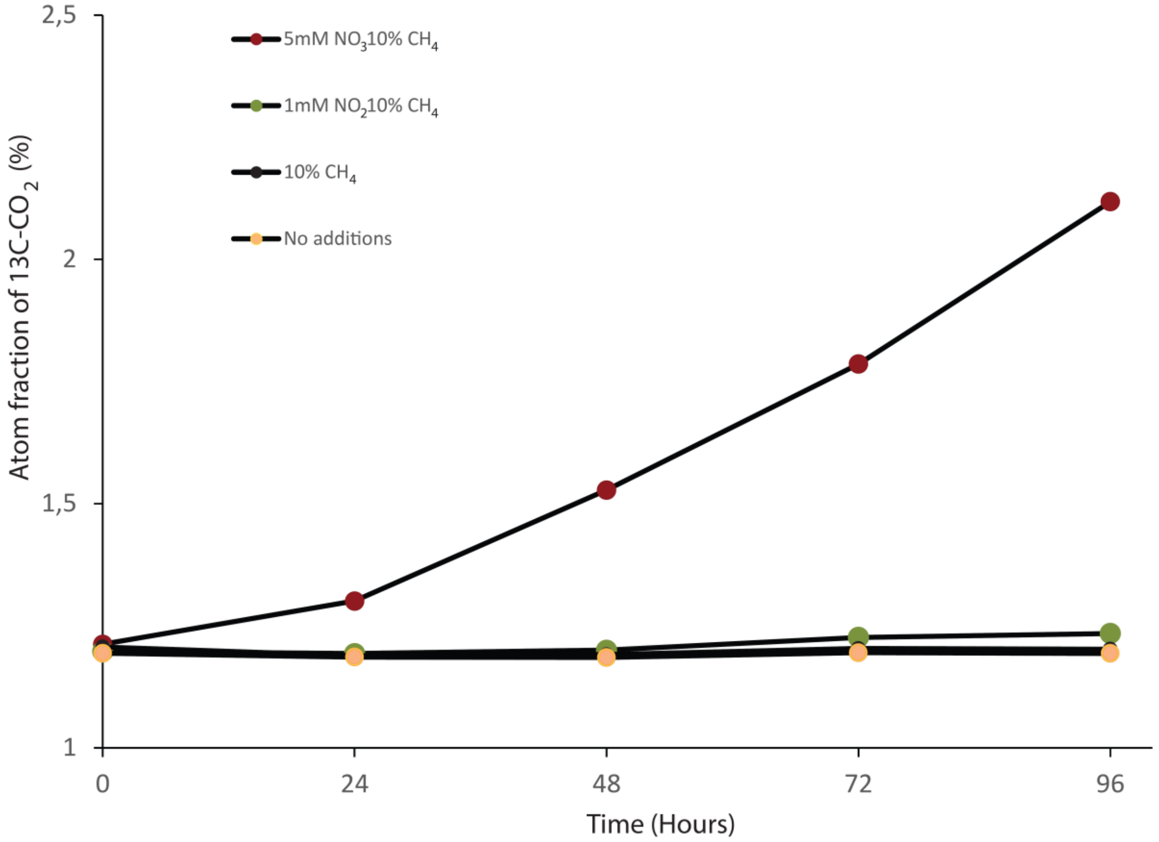

Supplementary Table S1: Summary of metagenomic sequencing after 1 and 2 years of enrichment.

Characteristic	Metagenome of 1 year	Metagenome of 2years
Number of raw reads generated	2721934	4044363
Average read length	261.8	190.6
Number of reads after trimming 100bp	2378934	2918314
Average read length after trim	286.7	231.5
Nr of reads mapped to Silva 123 truncated	1033	1425
% of reads assigned as 16S rRNA gene	0.04	0.05
Nr of reads aligned by SINA (70% cutoff)	1016	1423
Nr of reads classified by SILVA NGS	1014	1423

Supplementary Figure S2: Bin plot of the distribution of contigs in the pooled metagenomic reads at 1 and 2 years. The contigs marked in red were binned for the genome assembly of '*Candidatus* Methanoperedens nitroreducens Vercelli'.

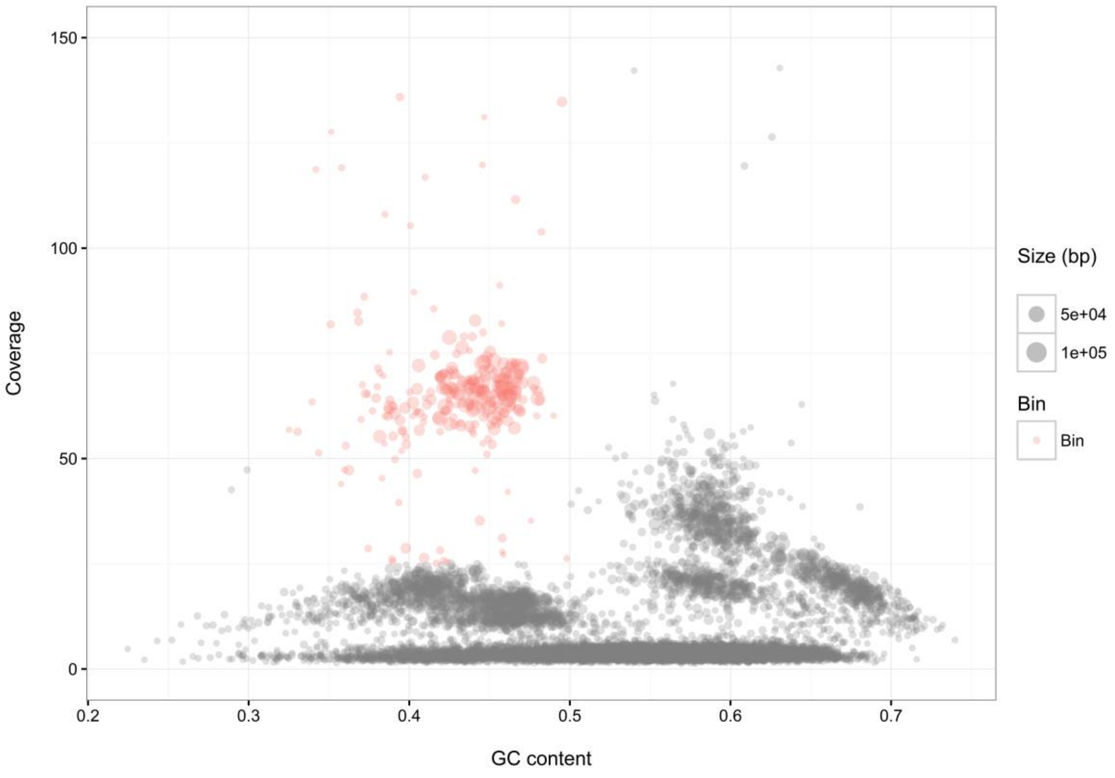

Supplementary Table S2: Analysis of the genome of '*Candidatus* Methanoperedens nitroreducens Vercelli'. The nucleotide sequences of the key enzymes in the methane oxidation and nitrate reduction pathways were compared to those of '*Candidatus* Methanoperedens sp. BLZ1 MPEBLZ' and '*Candidatus* Methanoperedens nitroreducens strain ANME-2d'.

**Methane oxidation**	Gene	locus_tag	Candidatus Methanoperedens sp. BLZ1 MPEBLZ	Candidatus Methanoperedens nitroreducens strain ANME-2d	Nuc. Identity to MPEBLZ (%)	Nuc. Identity to ANME-2d (%)
Methyl coenzyme M reductase subunit A	mcrA	MNV_v2_40019	MPEBLZ_01201	ANME2D_01104	85	92
Methyl-coenzyme M reductase subunit G	mcrG	MNV_v2_40018	MPEBLZ_01202	ANME2D_01103	83	90
Methyl-coenzyme M reductase subunit B	mcrB	MNV_v2_40016	MPEBLZ_01204	ANME2D_01101	83	88
Methyl-coenzyme M reductase operon protein D	protein D	MNV_v2_210057	MPEBLZ_01203	ANME2D_01102	78	84
Methyl-coenzyme M reductase protein C	proteinC	MNV_v2_200011	MPEBLZ_03729	ANME2D_00875	77	78
Methenyltetrahydromethanopterin cyclohydrolase	Mch	MNV_v2_210002	MPEBLZ_00120	ANME2D_02789	79	83
F420-dependent methylene –H4MPT reductase	Mer	MNV_v2_410015	MPEBLZ_02423	ANME2D_02259	81	83
F420-dependent methylene H4MPT dehydrogenase	Mtd	MNV_v2_820015	MPEBLZ_02677	ANME2D_01636	81	78
tetrahydromethanopterin S-methyltransferase subunit MtrH	MtrH	MNV_v2_470001	MPEBLZ_02584	ANME2D_00495	83	79
tetrahydromethanopterin S-methyltransferase subunit MtrG	MtrG	MNV_v2_470002	MPEBLZ_02585	ANME2D_00494	81	78
tetrahydromethanopterin S-methyltransferase subunit MtrF	MtrF	MNV_v2_470003	MPEBLZ_02586	ANME2D_00493	73	79
tetrahydromethanopterin S-methyltransferase subunit MtrA	MtrA	MNV_v2_470004	MPEBLZ_02587	ANME2D_00492	78	82
tetrahydromethanopterin S-methyltransferase subunit MtrB	MtrB	MNV_v2_470005	MPEBLZ_02588	ANME2D_00491	75	78
tetrahydromethanopterin S-methyltransferase subunit MtrC	MtrC	MNV_v2_470006	MPEBLZ_02589	ANME2D_00490	75	77
tetrahydromethanopterin S-methyltransferase subunit MtrD	MtrD	MNV_v2_470007	MPEBLZ_02590	ANME2D_00489	75	75
tetrahydromethanopterin S-methyltransferase subunit MtrE	MtrE	MNV_v2_470008	MPEBLZ_02591	ANME2D_00488	81	78
Tungsten dependent Formylmethanofuran dehydrogenase subunit C	FwdC	MNV_v2_430010	MPEBLZ_01216	ANME2D_01681	79	78
Formylmethanofuran dehydrogenase subunit A	FwdA	MNV_v2_430009	MPEBLZ_01217	ANME2D_01680	80	83
Tungsten dependent Formylmethanofuran dehydrogenase subunit B	FwdB	MNV_v2_430008	MPEBLZ_01218	ANME2D_01679	76	81
Formylmethanofuran dehydrogenase subunit D	FwdD	MNV_v2_430007	MPEBLZ_01219	ANME2D_01678	77	80
Tungsten dependent Formylmethanofuran dehydrogenase subunit E	FwdE	MNV_v2_470027	MPEBLZ_01782	ANME2D_00408	73	75
Formylmethanofuran dehydrogenase subunit B	FwdB	MNV_v2_330058	MPEBLZ_04482	ANME2D_01941	73	77
Formylmethanofuran dehydrogenase subunit G	Fwd subunit G	MNV_v2_330057	MPEBLZ_04483	ANME2D_01942	77	81

**$F_{420}H_2$dehydrogenase (Fpo)**						
$F_{420}H_2$ dehydrogenase subunit FpoA	FpoA	MNV_v2_110018	MPEBLZ_00739	ANME2D_00974	80	86
$F_{420}H_2$ dehydrogenase subunit FpoB	FpoB	MNV_v2_110017	MPEBLZ_00738	ANME2D_00973	78	83
$F_{420}H_2$ dehydrogenase subunit FpoC	FpoC	MNV_v2_110016	MPEBLZ_00737	ANME2D_00972	75	78
$F_{420}H_2$ dehydrogenase subunit FpoD	FpoD	MNV_v2_110015	MPEBLZ_00736	ANME2D_00971	76	80
$F_{420}H_2$ dehydrogenase subunit FpoH	FpoH	MNV_v2_110013	MPEBLZ_00733	ANME2D_00970	74	80
$F_{420}H_2$ dehydrogenase subunit FpoI	FpoI	MNV_v2_110012	MPEBLZ_00732	ANME2D_00969	72	81
$F_{420}H_2$ dehydrogenase subunit FpoJ_2	FpoJ2	MNV_v2_110011	MPEBLZ_00731	ANME2D_00968	80	79
$F_{420}H_2$ dehydrogenase subunit FpoJ_1	FpoJ1	MNV_v2_110010	MPEBLZ_00730	ANME2D_00967	74	78
$F_{420}H_2$ dehydrogenase subunit FpoK	FpoK	MNV_v2_2460007	MPEBLZ_00729	ANME2D_00966	99	82
$F_{420}H_2$ dehydrogenase subunit FpoL	FpoL	MNV_v2_2460008	MPEBLZ_00728	ANME2D_00965	99	73
$F_{420}H_2$ dehydrogenase subunit FpoM	FpoM	MNV_v2_2460009	MPEBLZ_00741	ANME2D_00964	100	79
$F_{420}H_2$ dehydrogenase subunit FpoN	FpoN	MNV_v2_2460010	MPEBLZ_00742	ANME2D_00963	100	72
$F_{420}H_2$ dehydrogenase subunit FpoO	FpoO	MNV_v2_2460011	MPEBLZ_00743	ANME2D_00962	99	74
$F_{420}H_2$ dehydrogenase subunit FpoF	FpoF	MNV_v2_410016	MPEBLZ_02422	ANME2D_02258	76	80
**Energy-conserving hydrogenase (Ech)**						
Energy-conserving hydrogenase subunit EchA	EchA	MNV_v2_700067	MPEBLZ_04052	ANME2D_02724	70	73
Energy-conserving hydrogenase subunit EchB	EchB	MNV_v2_700068	MPEBLZ_04051	ANME2D_02723	74	78
Energy-conserving hydrogenase subunit EchC	EchC	MNV_v2_700073	MPEBLZ_04043	ANME2D_02718	80	83
Energy-conserving hydrogenase subunit EchE	EchE	MNV_v2_700071	MPEBLZ_04046	ANME2D_02720	73	78
Energy-conserving hydrogenase subunit EchF	EchF	MNV_v2_700072	MPEBLZ_04044	ANME2D_02719	71	77
**Cytoplasmic Heterodisulfide reductase (Hdr)**						
cytoplasmic heterodisulfide reductase subunit HdrC_1	HdrC1	MNV_v2_1000002	MPEBLZ_01151	ANME2D_02156	78	79
cytoplasmic heterodisulfide reductase subunit HdrB_1	HdrB1	MNV_v1_150019	MPEBLZ_01152	ANME2D_02157	79	78
cytoplasmic heterodisulfide reductase subunit HdrA_1	HdrA1	MNV_v2_1000006	MPEBLZ_01153	ANME2D_02158	81	81
cytoplasmic heterodisulfide reductase subunit HdrC_3	HdrC2	MNV_v2_1040008	MPEBLZ_03074	ANME2D_03125	73	75
cytoplasmic heterodisulfide reductase subunit HdrB_3	HdrB2	MNV_v2_1040009	MPEBLZ_03073	ANME2D_03124	75	77
cytoplasmic heterodisulfide reductase subunit HdrA_3	HdrA2	MNV_v2_1000004	MPEBLZ_01155	ANME2D_02160	79	80
cytoplasmic heterodisulfide reductase subunit HdrC_1	HdrC3	MNV_v2_20034	MPEBLZ_01258	ANME2D_02551	74	76
cytoplasmic heterodisulfide reductase subunit HdrB_1	HdrB3	MNV_v2_20033	MPEBLZ_01259	ANME2D_02552	75	79
cytoplasmic heterodisulfide reductase subunit HdrA_1	HdrA3	MNV_v2_20032	MPEBLZ_01260	ANME2D_02553	79	80

**Membrane bound Hdr**						
membrane-bound heterodisulfide reductase subunit HdrD	HdrD	NA	MPEBLZ_01018	ANME2D_02796		
membrane-bound heterodisulfide reductase subunit HdrE	HdrE	NA	MPEBLZ_01017	ANME2D_02797		
**F420reducing hydrogenase (Frh)**						
F420-reducing hydrogenase subunit FrhB	FrhB	MNV_v2_1000008	MPEBLZ_01158	ANME2D_02162	80	83
Formylmethanofuran--tetrahydromethanopterin formyltransferase	Ftr	MNV_v2_1070031	MPEBLZ_03394	ANME2D_00639	77	81
**Nitrogen metabolism**						
Nitrite oxidoreductase/nitrate reductase alpha subunit	NarG	MNV_v2_560081	MPEBLZ_02035	ANME2D_03460	78	76
Nitrate reductase Beta subunit	NarH	MNV_v2_560080	MPEBLZ_02036	ANME2D_03461	79	80
Nitrite oxide heme protein	NirJ2/NirD	MNV_v2_370033	MPEBLZ_01277	ANME2D_02630	72	79
Nitric oxide reductase large subunit	NorB	MNV_v2_560086	MPEBLZ_01120	NA	83	NA
Nitric oxide reductase	FprA	MNV_v2_120012	MPEBLZ_01124	ANME2D_01839	67	78
Nitrous oxide reductase	NosZ	MNV_v2_560077	MPEBLZ_02039	ANME2D_03464	74	81
Nitrogenase iron protein	nifH	MNV_v2_630004	MPEBLZ_00580	ANME2D_00769	77	82
Nitrogenase subunit alpha	nifD	MNV_v1_540009	MPEBLZ_00583	ANME2D_00766	83	80

Supplementary Table 2: Analysis of the genome of '*Candidatus* Methanoperedens nitroreducens Vercelli', the nucleotide identity of the key enzymes of the methane oxidation and nitrate reduction pathway are compared to '*Candidatus* Methanoperedens sp. BLZ1 MPEBLZ' and '*Candidatus* Methanoperedens nitroreducens strain ANME-2d'.
